# Delayed Diagnosis and Treatment of Traumatic Testicular Dislocation: A Case Report and Literature Review

**DOI:** 10.3389/fsurg.2021.721192

**Published:** 2021-10-04

**Authors:** Qihua Wang, Rami W. A. Alshayyah, Hang Lv, Yang Yu, Xinyu Liu, Bo Yang

**Affiliations:** Department of Urology, The Second Hospital of Dalian Medical University, Dalian, China

**Keywords:** testicular trauma, testicular dislocation, case report, urethral injury, delayed diagnosis

## Abstract

Traumatic testicular dislocation is a rare complication secondary to different kinds of accidents. A 61-year-old man, who was injured by wall collapse and was diagnosed as pelvic fracture and posterior urethral rupture 5 months ago, came to the urologic department to seek urethral reconstruction. However, thorough physical examination and imaging examination confirmed a round mass in the right inguinal region and an empty right scrotum, which support diagnosis of testicular dislocation. The patient did not take the initiative to complain about that because he thought the right testis had been destroyed by the accident already. So the patient underwent fiber cystourethroscopy, urethral reconstruction, and orchiopexy. No testicular atrophy was confirmed at follow-up. We reviewed previous reports about traumatic testicular dislocation and analyzed the cause of delayed diagnosis.

## Case Presentation

A 61-year-old man went through a pelvic fracture due to wall collapse and was diagnosed with posterior urethral rupture. Five months after the accident, he came to our department to seek for urethral reconstruction.

**Figure 1:**
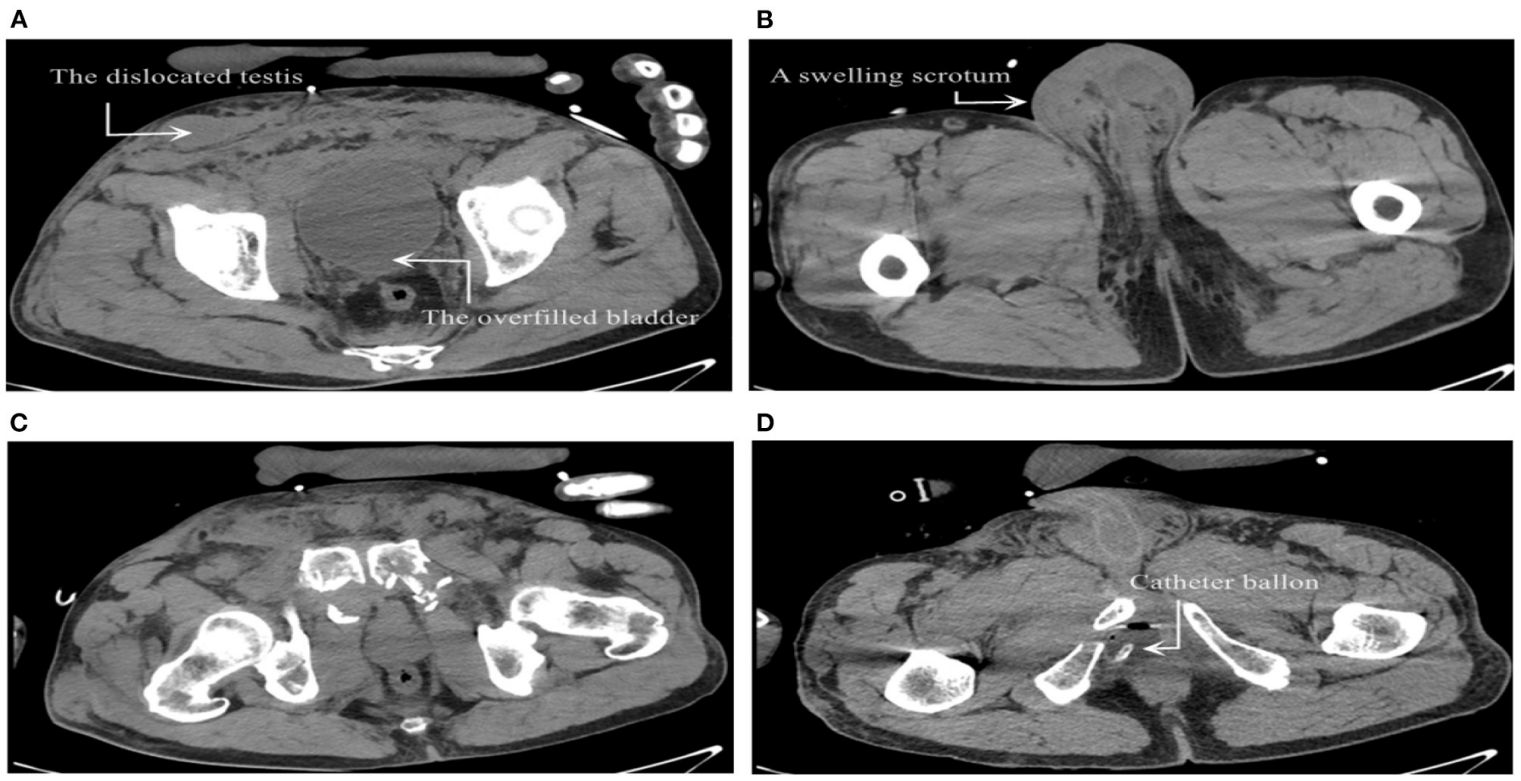
**(A)** The right testis was located in the subcutaneous fat layer and the bladder was overfilled. **(B)** Obvious swelling of scrotum, and no testicles were found in the right scrotum. **(C)** Comminuted fracture of pelvis. **(D)** The catheter balloon was outside the bladder.

We retrieved his medical history after injury from the medical record system in the orthopedic department. After the injury, the patient went through acute urinary retention (Figure 1A). Computed tomography (CT) scan showed edematous lower limbs and perineum (Figure 1B), purpura, and obvious local tenderness. The condition of the scrotum and testicles was not recorded. CT scan showed a comminuted fracture of the pelvis and sacrum (Figure 1C). The catheter balloon was not located in the bladder (Figure 1D). At that time, the orthopedists asked a urologist for a consultation on the acute urinary retention of the patient. The patient was then diagnosed as having a pelvic fracture complicated with acute urinary retention caused by posterior urethral rupture. Cystostomy and external pelvic fixation were performed.

The patient then came to the urology department to seek for urethral reconstruction 5 months after injury. Physical examination revealed a tough, round palpable mass in his right inguinal region, with a size of about 4^*^2 cm. The right scrotum was shrunken whereas the other side was normal. After further questioning the medical history, the patient confirmed that his bilateral testicles were palpable in scrotum and denied history of retractile testes before the accident, and the right scrotum was found empty after he was discharged from the hospital last time. He supposed the testicle had been completely destroyed during the accident. The patient was old, had no fertility requirements, and still had erectile function, so he did not take the initiative to mention it to his doctor.

**Figure 2:**
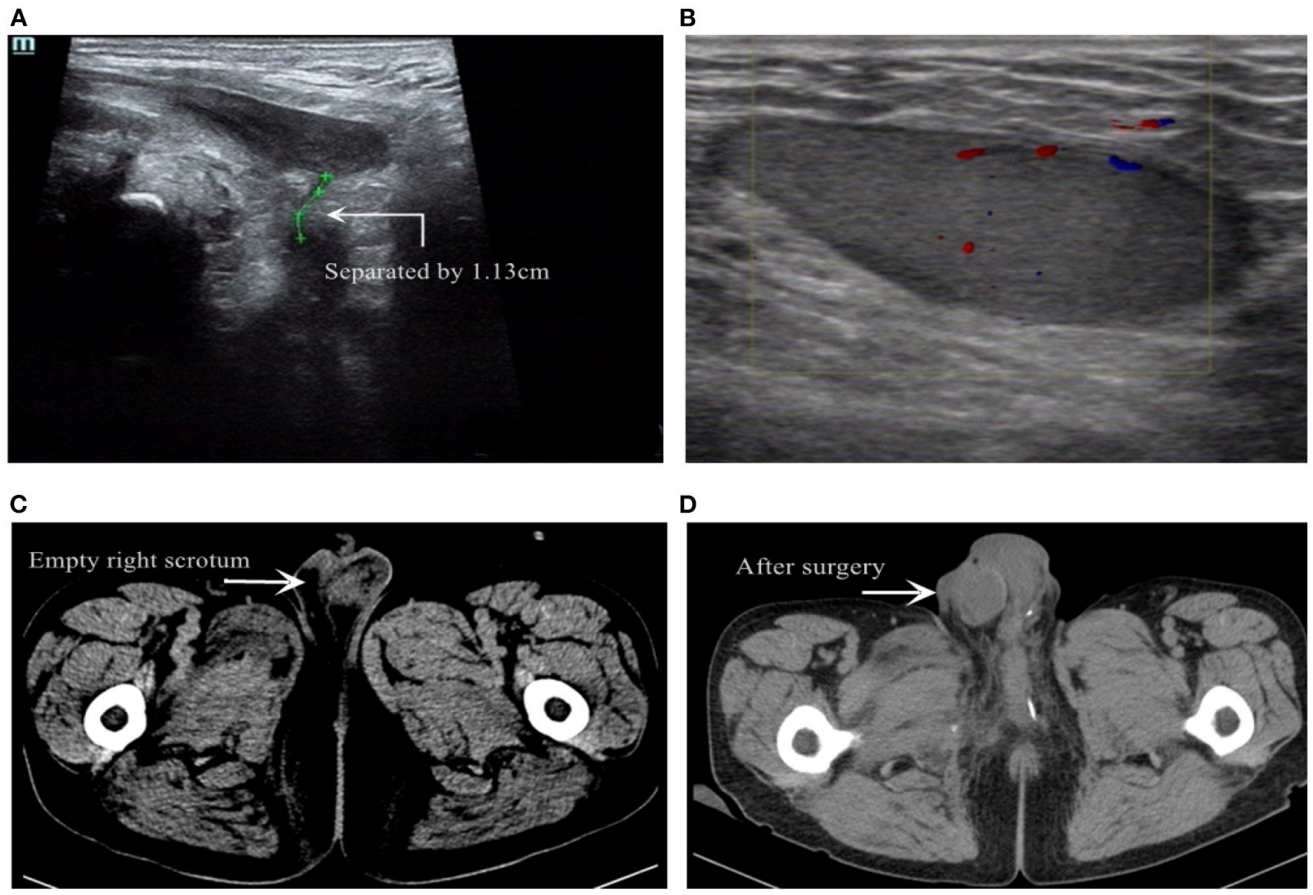
**(A)** Transpelvic ultrasound examination revealed membranous urethral rupture. **(B)** Color Doppler US image of the inguinal mass revealed a intact shape of testicle with blood supply. **(C)** Pelvic CT showed the swollen scrotum and the empty right scrotum. **(D)** Re-examination of pelvic CT after surgery.

Pelvic floor ultrasonography (Figure 2A) was done during transurethral injection of normal saline, and presented that the penile part of the urethra was naturally dilated and the mucosa was smooth. However, the membranous urethra was like the shape of the mouth of a bird. There was no dilatation of membranous, bulbar, and prostatic urethra (indicating posterior urethral rupture). Color Doppler ultrasound image of right inguinal mass (Figure 2B) presented a moderate echo with a size of 4.1^*^1.2 cm in the subcutaneous fat layer, with clear boundary, intact shape, and blood flow signals, which was consistent with a normal testicular echo. Reexamination of the pelvic CT showed that the right scrotum was empty and asymmetrical to the left (Figure 2C). The patient was diagnosed with membranous urethral rupture and right traumatic testicular dislocation.

After adequate antiinfection treatment, fiber cystourethroscopy, urethral reconstruction, and orchiopexy for the dislocated testicle were performed under general anesthesia. The posterior urethral atresia was confirmed by fiber endoscopy through the external urethral orifice and cystostomy catheter. An invert “Y” incision was made in the perineum. Then the urethra was exposed and separated. Then we excised the scar tissue around the urethra, and the defect length was measured at about 1 cm. After eight stitches of tension-free anastomosis of the proximal and distal urethra, the incision was sutured. Negative pressure drainage was retained. Then, an oblique incision of about 3 cm was made in the right inguinal region, and a well-formed dislocated testicle was exposed outside the subcutaneous inguinal ring with no rupture of the tunica albuginea and any hematoma. We separated the spermatic cord and vas deferens, pulled the testicles out of the scrotum, and fixed them with three stitches to prevent from torsion.

## Outcome and Follow-Up

One month after the operation, scrotal ultrasound showed well-blood supply for bilateral testicular and no sign of atrophy. CT re-examination (Figure 2D) confirmed the testicles remained in place. Also, the patient claimed to have a satisfactory erectile function.

## Discussion

Pelvic fracture is usually caused by intense energy trauma, and its mortality rate reaches 19% (1). In the case under the condition of unstable hemodynamics, the mortality rate can be up to 37% (2). Around 5.34% of men and 3.62% of women with pelvic fractures in the National Trauma Database of the United States (*n* = 31,380) were complicated with urogenital system injuries (3), such as rupture of the bladder, corpus cavernosum, and urethra. Among them, male urethral injury is more common than female urethral injury (1.54 vs. 0.15%) (3). But the possibility of testicular dislocation is not specifically mentioned. The injury of the posterior urethra can cause serious urine extravasation, which may penetrate into the retropubic space and around the bladder. In addition, extensive swelling and ecchymosis often make it difficult for clinicians to fully investigate the injury and even delay diagnosis and treatment.

Traumatic testicular dislocation, as a very rare complication (4), is rarely reported accompanied by pelvic fracture. Subramaniam et al. (5) reviewed published articles and only 180 cases (243 testicles) were reported in the past two centuries (from 1936 to 2017). According to previous reports (3, 6), it could be caused by traffic accidents (motorcycles, motor vehicles, etc.), riding injuries, kicking injuries, explosion injuries, etc. The most classic cause is motorcycle accidents (5), when sudden deceleration leads the scrotum to strike against the fuel tank and dislocate testes, whereas in this case, the injury was due to the collapse and crushing of the wall during the work of smashing the wall. Besides, there were also some reports describing the delayed diagnosis of penile dislocation, which could be dislocated into scrotum (7, 8) or in the foreside of pubic bone (9, 10). In this situation, the patient will have urine retention (8), which should be differentiated from urethral rupture secondary to pelvic fracture.

After the injury, some patients suffered from direct testicular trauma (3) or secondary testicular torsion (11), which may lead to loss of blood supply and even severe necrosis. However, it is more rare that testes may be dislocated in the shallow or deep space, unilateral or bilateral, due to direct mechanical force or strong cremasteric reflex under stress (12). The most common site is superficial inguinal ring (13). Dislocated testes may reach pubic symphysis, inguinal canal, and even retroperitoneum (6).

Delayed diagnosis makes dislocated testes stagnate at a higher temperature than the scrotum (about 4°C) for a long time (14). Thus, complications similar to cryptorchidism may occur, including testicular atrophy, hypofunction, torsion, and even testicular malignant tumor (14, 15), which causes disability and medical disputes. In many recent reports on traumatic testicular dislocation, the diagnosis was often delayed for several weeks or even years before treatment (6). There was even a report that the diagnosis of bilateral testicular dislocation was delayed 15 years after azoospermia (16). Marvelously, his spermatogenesis slowly recovered post-operatively and the couple obtained a healthy child by natural sexual intercourse 40 months after surgery (16).

A study from China investigated 1,967 male patients who experienced blunt abdominal trauma within 15 years (6). Nine patients were finally diagnosed as testicular dislocation, and all of them were initially missed diagnoses. Only seven cases had typical CT findings, whereas only three cases got timely CT diagnosis of testicular dislocation. After closed manual reduction, these three patients avoided surgery. However, among the patients whose testicular dislocation was not reported by CT examination, two patients were later diagnosed by ultrasound. Among the six patients with delayed diagnosis (3–60 days, average 19 days), five patients underwent orchiopexy and one patient underwent orchiectomy due to testicular torsion and infarction (6).

We analyzed the causes of this delayed diagnosis. First of all, the pelvic fracture is often so critical that the focus of treatment often needs to be placed on aspects of life support, fluid resuscitation, bleeding control, whereas some inconspicuous non-critical diseases may be ignored. The patients often presented with severe pain, local ecchymosis, and swelling, which is easy to cover up the underlying situation. On the other hand, clinicians and imaging experts are likely to pay more attention to important organs when watching images during a period of emergency, and are eager to report important results faster, which leads to incomplete analysis. The overreliance on imaging reports may lead to neglect the importance of physical examination, and the physical examination of the reproductive system is easily neglected and shameful to carry out. In addition, with the specialization of functions in different departments of the hospital, for patients hospitalized in other departments, consultants may be lacking in communication and physical examination, which leads to delayed diagnosis. Patients misunderstand that their own state of illness will also lead to delayed diagnosis.

## Limitations

Our report does not involve any photos of the affected area and the surgery situation because we lost the photos. We could recreate the injury situation of the day of the patient by having retrieved his medical record and previous CT images. Some references are outdated because traumatic testicular dislocation is a rare complication and lacks authoritative guidelines.

## Conclusions

With the development of medical technology, patients with severe injuries have more chances of survival. However, we should not neglect the complications and sequelae that are not fatal, but may cause serious consequences. As a rare complication, concealed traumatic testicular dislocation secondary to pelvic fracture is easily missed and can cause complications like testicular atrophy, hypofunction, torsion, and even testicular malignant tumor (6, 14, 15). Some patients may be delayed for a long time, even after 15 years (16). Correct identification and treatment can prevent some complications such as testicular atrophy, and improve or even reverse other complications such as azoospermia (16). More clinicians should be aware of and value this so as to avoid delayed diagnosis and reduce the disability rate of patients.

## Data Availability Statement

The original contributions presented in the study are included in the article/supplementary material, further inquiries can be directed to the corresponding author/s.

## Ethics Statement

The studies involving human participants were reviewed and approved by the Second Affiliated Hospital of Dalian Medical University. The patients/participants provided their written informed consent to participate in this study. Written informed consent was obtained from the individual(s) for the publication of any potentially identifiable images or data included in this article.

## Author Contributions

BY and YY: protocol and project development. QW: data collection and management. QW and HL: manuscript writing and editing. All authors contributed to the article and approved the submitted version.

## Conflict of Interest

The authors declare that the research was conducted in the absence of any commercial or financial relationships that could be construed as a potential conflict of interest.

## Publisher's Note

All claims expressed in this article are solely those of the authors and do not necessarily represent those of their affiliated organizations, or those of the publisher, the editors and the reviewers. Any product that may be evaluated in this article, or claim that may be made by its manufacturer, is not guaranteed or endorsed by the publisher.
